# In vitro production of desired sex ovine embryos modulating polarity of oocytes for sex-specific sperm binding during fertilization

**DOI:** 10.1038/s41598-022-09895-2

**Published:** 2022-04-07

**Authors:** Ramesh Kumar G., Ashish Mishra, Arindam Dhali, Ippala Janardhan Reddy, Debpriyo Kumar Dey, Dintaran Pal, Raghavendra Bhatta

**Affiliations:** ICAR-National Institute of Animal Nutrition and Physiology, Adugodi, Bengaluru, 560 030 India

**Keywords:** Embryology, Animal biotechnology

## Abstract

The present study aimed to modulate the oxidative status-mediated polarity of the oocytes for sex-specific sperm fertilization to generate desired sex embryos. In vitro embryos were produced at different oxidative status, varying O_2_ concentrations, and without/with l-carnitine in maturation and culture media. The majority of the embryos produced at high oxidative stress were males whereas; low oxidative status favoured female embryos production. Low O_2_ doubled the proportion of female embryos (10.59 vs 21.95%); however, l-carnitine supplementation in media increased approximately seven-folds of the female embryos (12.26 vs. 77.62%) production. Oocytes matured at high oxidative status were in the repolarized state favouring positively charged Y sperm fertilization to produce significantly more male embryos. Low oxidative status favoured negatively charged X sperm fertilization to the oocytes in the depolarized state to produce more female embryos. Intracellular ROS was significantly low in female embryos than in males; however, female embryos were more stressful than males*.* The study concluded that the oxidative status-mediated alteration in pH of the medium to modulate the intracellular positive ions is the main critical factor to influence the sex of embryos through sex-specific sperms fertilization to the oocytes as per their polarity.

## Introduction

**Figure 1:**
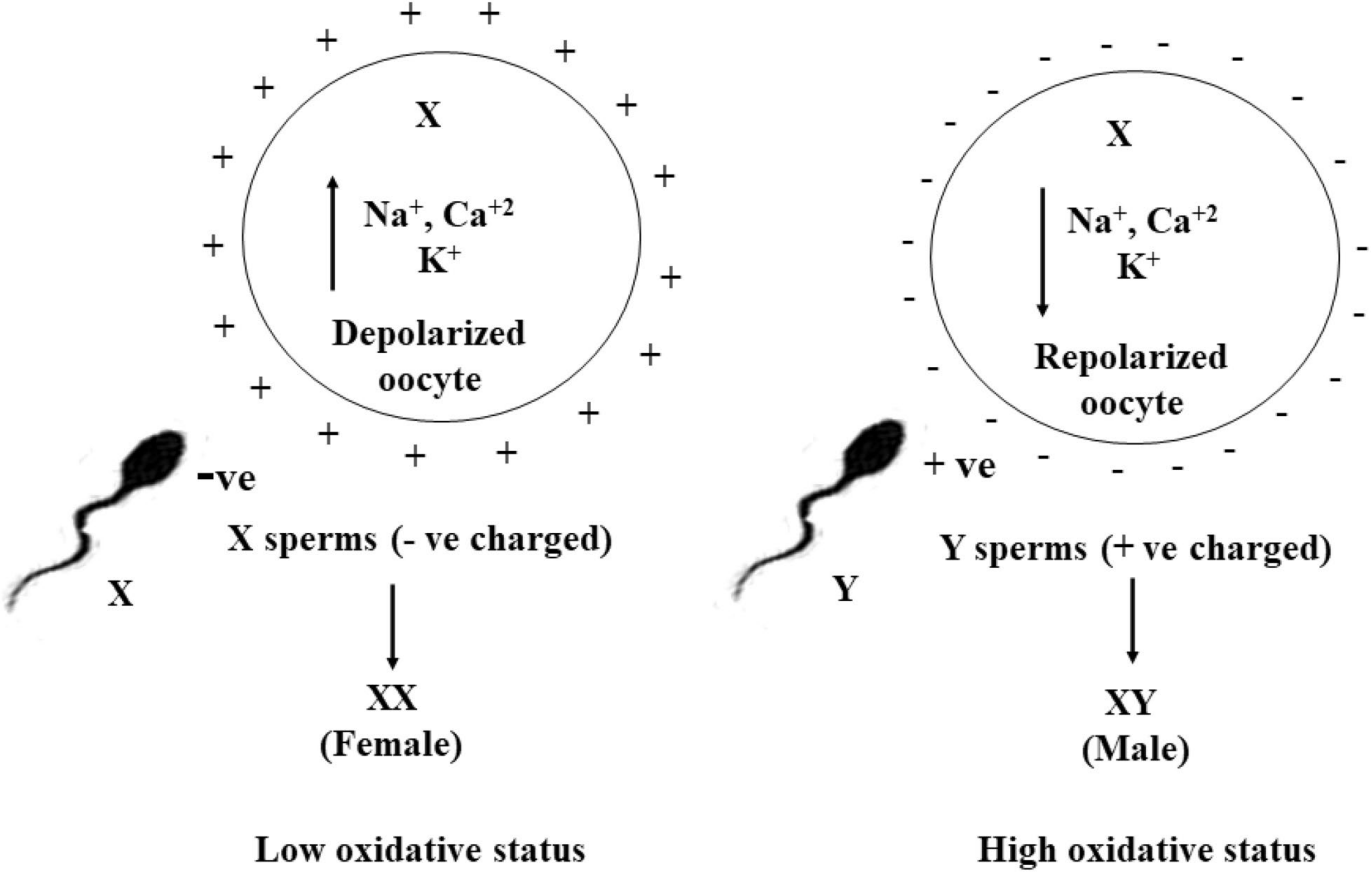
Oxidative status mediated change in polarity of oocytes for sex-specific sperm fertilization to produce selected sex offsprings.

The success rate of in vitro embryo production (IVEP) has been improved over time to produce more quality embryos, employing various culture media^1,2^. Scientific interest for today’s embryonic research is to produce quality embryos in vitro; however, our target is to produce sex-specific embryos. Globally, many methods are reported for sperm sorting to get selected sex calves; however, most of them need scientific validation. Therefore, an alternative approach to sex-sorted semen is in vitro production of sexed embryos. There are many factors, i.e., temperature, gas composition (O_2_/CO_2_), pH, media composition, and air quality, responsible for the oxidative status of the culture system and affect the developmental potential of embryos. However, ambiguity still exists, and no such report is available to know the impact of culture condition mediated oxidative stress on sex-specific embryo production and their developmental potential in vitro. Despite the abundance of case reports on oxidative stress-mediated embryonic sex bias in humans and mice both in vitro and in vivo, scanty reports are available on animal studies. High O_2_ concentration is more detrimental to female embryos than males. Male embryos develop faster than females in vitro, but the reverse is in vivo^3^. Suboptimal culture conditions result in loss of female embryos in vitro and induce deviations in gene expression more in male embryos than in females^4,5^. The sex ratio skewed towards males in summer and females in winter^6^. Increased levels of reactive oxygen species (ROS) inactivate energy metabolism, showing growth rate differences in male and female embryos^7^. Female embryos exhibit a fourfold higher activity of the pentose phosphate pathway than male^8^. The sex-specific difference in the developmental potential of embryos in specific conditions might be due to ROS-mediated inactivation of important genes. We have recently reported that low O_2_ reduced intracellular oxidative status and improved the developmental potential of in vitro embryos^9^. However, no such report is available on ROS-mediated improvement in the percentage and developmental potential of sex-specific embryos. All the above conditions for sex biasness can be speculated that high oxidative status hinders the development of female embryos than males. Hence, lowering the micromilieu ROS level can improve the percentage of female embryos, and modulation of ROS level can skew the sex ratio of embryos. To clarify this hypothesis, the present study was undertaken in the sheep model to determine the impact of culture-mediated oxidative status on the sex ratio of in vitro embryos. The polarity or membrane potential of oocytes is maintained due to disparities in ion concentrations across the membrane (intra and extracellular component)^10^. The level of oxidative status of culture condition might be influencing the intracellular ions concentration of oocytes to change their polarity. Hence, the primary objective of the study is to modulate the oxidative status of culture conditions to change the polarity of oocytes for sex-specific sperm fertilization to produce selected sex embryos (Fig. 1).

Different levels of oxidative status during IVEP were created by different O_2_ concentrations and supplementation of l-carnitine as a free radical scavenger in maturation and culture media. l-Carnitine-mediated reduction in oxidative stress on embryonic developmental potential is reported in various species^11–13^ with our studies in sheep^14,15^. However, this is the first report on the impact of oxidative stress on sex-specific embryos production in vitro. l-Carnitine (3-hydroxy-4-*N*-trimethylammino butyrate, C_7_H_5_NO_3_, M.W.-161.2) is a water-soluble quaternary ammonium compound and vitamin-like naturally occurring substance that acts as an antioxidant that neutralizes the free radicals and protects the cell against oxidative stress-induced apoptosis^16^. The subsequent objective of the study was to find out the sex-specific difference in mRNA abundance of genes related to antioxidant {(glutathione peroxidase *(GPx),* Cu/Zn superoxide dismutase *(SOD1),* Mn superoxide dismutase *(SOD2),* and catalase (CAT)}, glucose metabolism {Glyceraldehyde 3-phosphate dehydrogenase *(GAPDH)*, Glucose-6-phosphate dehydrogenase *(G6PD),* and Hypoxanthine–guanine phosphoribosyl transferase *(HPRT)*, and apoptotic {B-cell lymphoma protein 2 (*BCL2*), BCL2-associated protein (*BAX*), Caspase3 (*CASP3*), proliferating cell nuclear antigen (*PCNA*), and tumor suppressor protein (*p53*)} pathways to analyze the sexual dimorphism in the embryonic developmental potential in relation to the oxidative status of culture condition.

## Results

### In vitro ovine embryo production

All the developmental stages of embryos (cleavage to blastocyst) produced at different oxidative status (experiment I–III) are detailed in Table 1. Overall, the embryos produced at different O_2_ concentrations (20 vs. 5%) (experiment I) showed no significant (P < 0.05) differences in the cleavage rate (62.93 vs. 64.61%), whereas 5% O_2_ produced significantly (P < 0.05) more morula (40.21 vs. 25.42%) and blastocyst (18.92 vs. 8.16%) than at 20%. l-Carnitine (10 mM) supplementation in maturation and culture media (experiment II) resulted in significantly (P < 0.05) higher cleavage (69.64 vs. 63.24%), morula (49.93 vs. 24.64%), and blastocyst (32.56 vs.8.92%), than the group with no l-Carnitine. l-Carnitine (10 mM) supplementation in media with different O_2_ concentrations (experiment III) resulted in significantly (P < 0.05) higher percentage of cleavage (69.28 vs.10.26%), morula (51.36 vs.2.86%) and blastocyst (34.34 vs. 0%) at atmospheric O_2_ than at 5% O_2_.

**Table 1:** Developmental stages of in vitro ovine embryos produced in different experiments.

Experiments	Groups	Oxygen (%)	l-Carnitine in maturation medium (mM)	l-Carnitine in culture medium (mM)	Oocytes cultured	Cleavage (%)	Morula (%)	Blastocyst (%)
I	1	20	–	–	272	62.93 ± 2.8	25.42 ± 2.9^a^	8.16 ± 1.1^a^
	2	5	–	–	257	64.61 ± 2.1	40.21 ± 2.3^b^	18.92 ± 2.98^b^
II	1	20	0	0	288	63.24 ± 1.9^a^	24.64 ± 2.3^a^	8.92 ± 0.98^a^
	2	20	10	10	263	69.64 ± 2.3^b^	49.93 ± 2.8^b^	32. 56 ± 2.7^b^
III	1	Atmospheric	10	10	255	69.28 ± 3.2^a^	51.36 ± 2.7^a^	34.34 ± 2.4^a^
	2	5%	10	10	368	10.26 ± 1.8^b^	2.86 ± 0.3^b^	0^b^

*All developmental stages were calculated from the number oocytes cultured. Percentage results are presented as mean ± S.E.M.

Different superscripts within rows of same column differ significantly at P < 0.05 between groups of each experiment.

### Polarity of the matured oocytes

The intracellular sodium (Na^+^), potassium (K^+^), and calcium (Ca^2+^) ions concentration were estimated in the matured oocytes to check the ions dependent polarity (depolarization/repolarization) for the sex-specific charged sperm (X/Y) fertilization resulting in embryonic sex biasness (Table 2). The concentrations of the intracellular Na^+^ ion (mg/L) was 46.46 and 64.89, K^+^ ion (mg/L) was 28.86 and 27.76 and Ca^2+^ ion (mg/L) was 0.49 and 1.67 in oocytes matured at 20 and 5% O_2_ respectively. The intracellular Na^+^ ion was non-significantly high, and the Ca^2+^ ion was significantly (P < 0.05) high in oocytes matured at 5% O_2_ than at 20%. Whereas, K^+^ ion concentration was similar in both the groups. The concentrations of the intracellular Na^+^ ion (mg/L) was 47.15 and 241.41, K^+^ ion (mg/L) was 29.06 and 26.28 and Ca^2+^ ion (mg/L) was 0.51 and 5.52 in oocytes matured without and with L-carnitine respectively. Intracellular Na^+^ and Ca^2+^ ions were significantly (P < 0.05) high in the oocytes matured with L-carnitine than without l-carnitine. However, the K^+^ ion concentration was similar in both groups.

**Table 2:** Intracellular ions (Na^+^, K^+^ and Ca^2+^) concentration of matured oocytes at different level of oxidative status.

Experiments	Experiment I		Experiment II	
	Oocytes matured at different O_2_ concentrations		Oocytes matured with l-carnitine (10 mM) at 20% O_2_	
Ions (mg/L)	20%O_2_	5%O_2_	Without l-carnitine	With l-carnitine
Sodium	46.46 ± 8.36	64.89 ± 12.3	47.15 ± 9.77^a^	241.41 ± 40.36^b^
Potassium	28.86 ± 2.67	27.76 ± 3.23	29.06 ± 1.04	26.28 ± 3.54
Calcium	0.49 ± 0.12^a^	1.67 ± .25^b^	0.51 ± 0.16^a^	5.52 ± 1.7^b^

*Different superscripts in the same row differ significantly at P < 0.05.

### Sexing of the ovine blood and embryos

**Figure 2:**
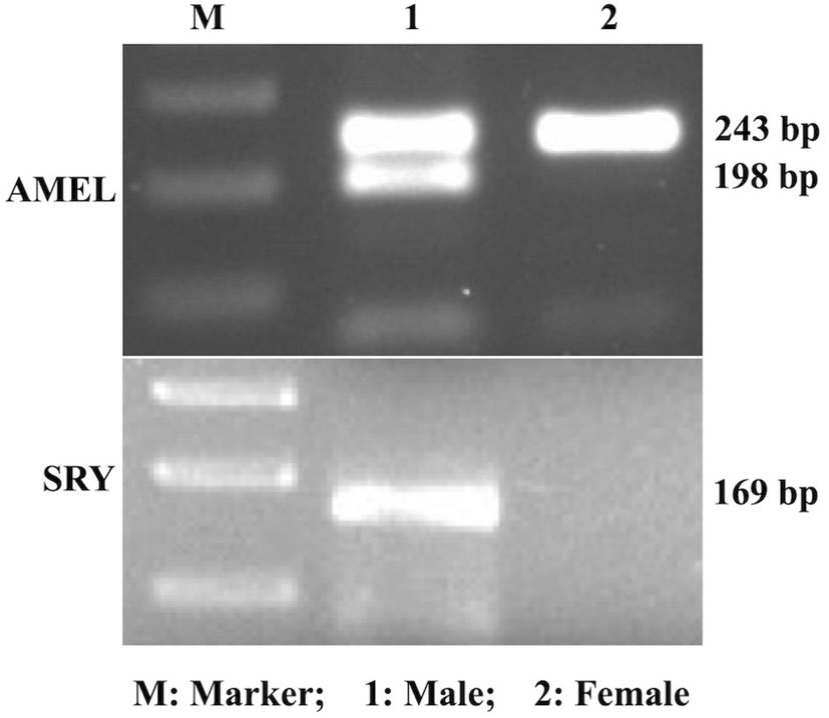
Expression of *AMEL* and *SRY* in genomic DNA of blood.

**Figure 3:**
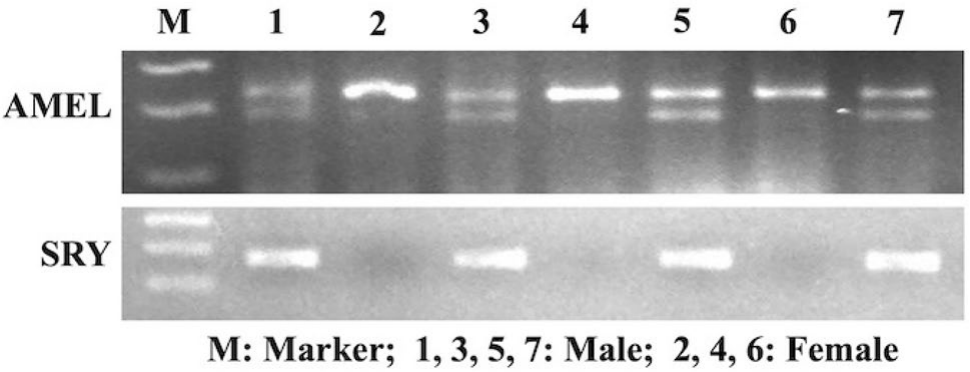
Expression of *AMEL* and *SRY* in genomic DNA of ovine embryos.

The sex of the blood and embryos was determined by PCR-based amplification of *AMEL* and *SRY* genes present in genomic DNA (gDNA). Sex-specific (male and female) blood was used as the positive sample in PCR for the primers to be used for sexing of embryos (Fig. 2, Supplementary Information). The PCR for amplification of the *AMEL* gene was in the form of two bands of 243 and 198 bp in males and a single band of 243 bp in females. However, amplification of the *SRY* gene was in the form of one band of 169 bp in males only, seen in gel electrophoresis to confirm the sex of embryos (Fig. 3, Supplementary Information).

### Sex ratio of embryos produced at different oxidative status

**Figure 4:**
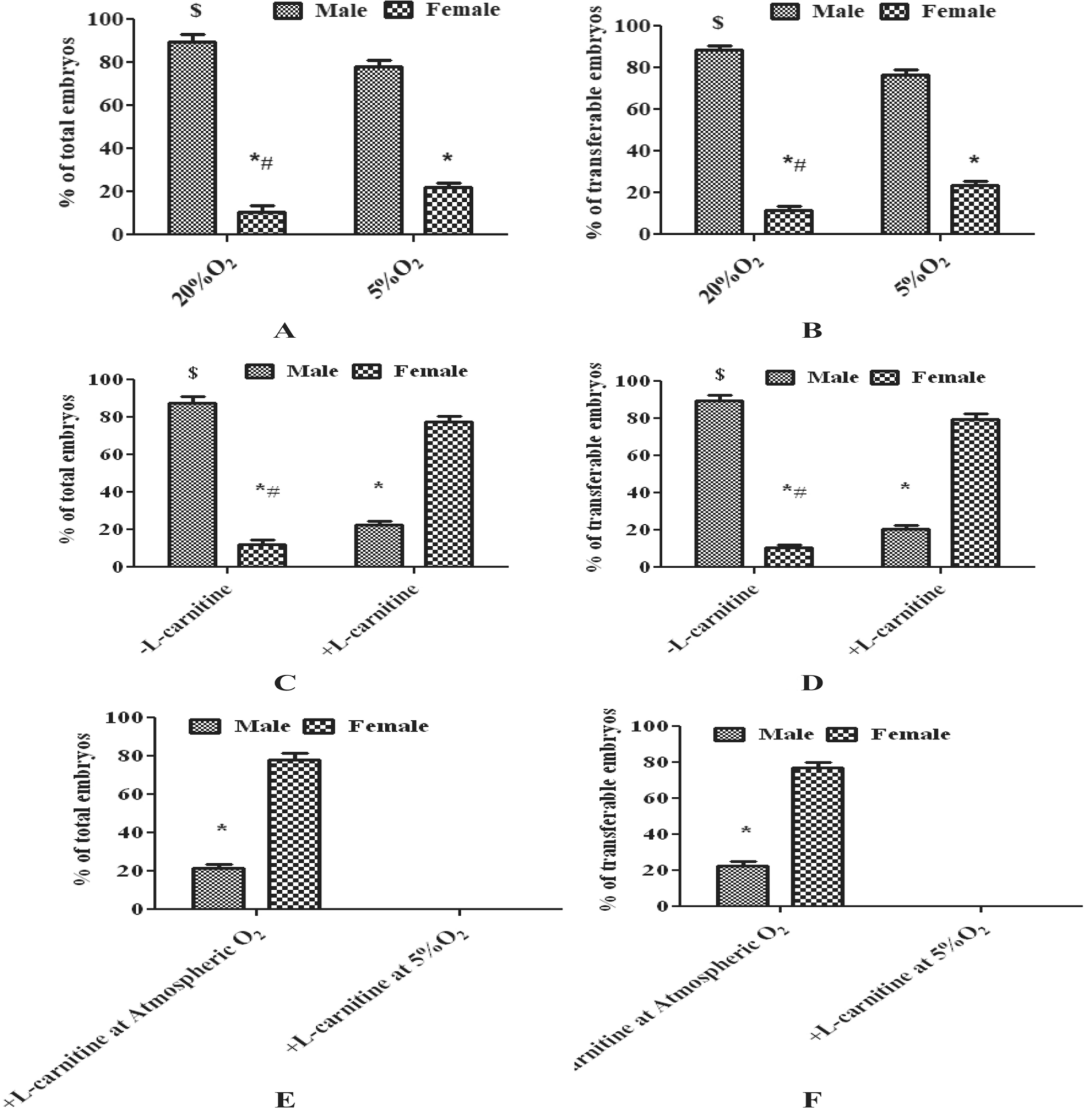
Sex ratio of the embryos produced in different experiments (I–III). (**A**) Sex ratio of the total embryos produced in experiment I. (**B**) Sex ratio of the transferable embryos produced in experiment I. (**C**) Sex ratio of the total embryos produced in experiment II. (**D**) Sex ratio of the transferable embryos produced in experiment II. (**E**) Sex ratio of the total embryos produced in experiment III. (**F**) Sex ratio of the transferable embryos produced in experiment III. *Significant difference between male and female of each group at P < 0.05. ^$^Significant difference between the males of both the groups at P < 0.05. ^#^Significant difference between the females of both the groups at P < 0.05.

All the embryos (cleavage to blastocyst) produced at different oxidative status (experiment I–III) were used for sexing except the embryos of experiment III, group 2. The embryos in all the experiments were grouped as total embryos (cleavage to blastocyst) and transferable embryos (morula and blastocyst). The sex ratio of embryos produced at different oxidative status is detailed in Fig. 4. 170 and 164 embryos produced from 20 and 5% O_2_ respectively (experiment I) were used for sex determination (Fig. 4A,B). The sex ratio of total embryos produced at 20% O_2_ was 89.41% male and 10.59% female and the transferable embryos were 88.54% male and 11.46% female. The total embryos produced at 5% O_2_ resulted in 78.05% male and 21.95% female and transferable embryos were 76.52% male and 23.48% female. Significantly (P < 0.05), more male embryos than females were produced at both the O_2_ concentrations. However, 20% O_2_ produced significantly (P < 0.05) more male embryos than at 5%. Whereas, significantly (P < 0.05), more female embryos were produced at 5%O_2_ than at 20%. 182 and 176 embryos produced at 20% O_2_ without and with l-carnitine supplementation respectively (experiment II) were used for sex determination (Fig. 4C,D). The sex ratio of total embryos produced without l-carnitine was 87.74% male and 12.26% female and the transferable embryos were 89.61% male and 10.39% female. Subsequently, l-carnitine supplementation in maturation and culture media resulted in total embryos of 22.38% male and 77.62% female and transferable embryos of 20.54% male and 79.46% female. Significantly (P < 0.05), more male embryos were produced without l-carnitine and L-carnitine supplementation resulted in significantly (P < 0.05) more female embryos. 174 and 52 embryos produced from atmospheric and 5% O_2_ respectively with l-carnitine supplementation (experiment III) were used for sex determination (Fig. 4E,F). The sex ratio of total embryos produced at atmospheric O_2_ supplemented with l-carnitine was 21.67% male and 78.33% female and the transferable embryos were 22.73% male and 77.27% female. l-Carnitine supplementation in the media at atmospheric O_2_ showed embryonic sex biasness toward females. However, the developmental stages of embryos were significantly low at 5% O_2_ supplemented with l-carnitine. Hence, the embryonic sex ratio of this group was not calculated.

### Intracellular ROS levels in sex-specific embryos

**Figure 5:**
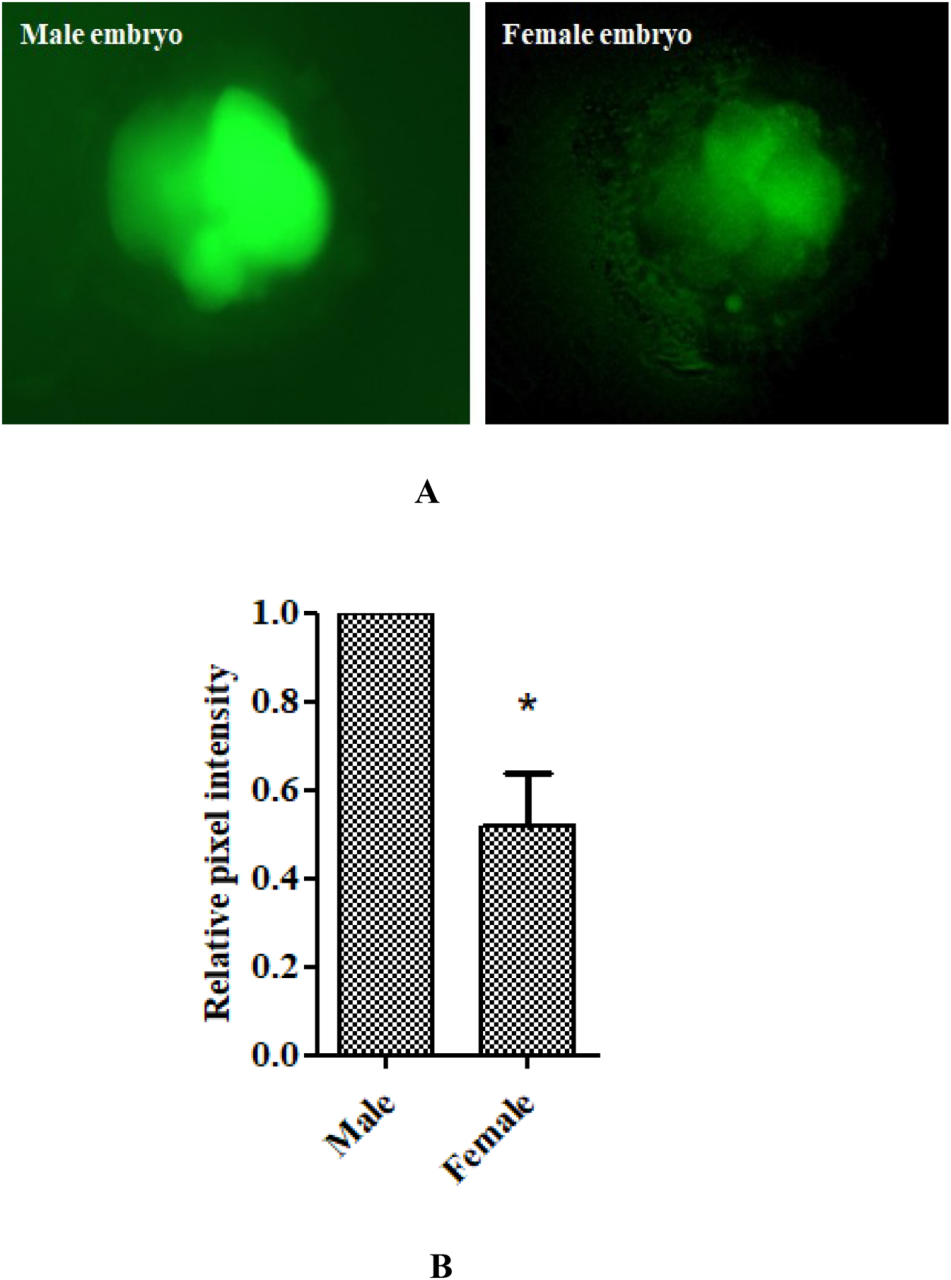
Sex-specific differences in intracellular embryonic ROS level. *Significant difference between male and female at P < 0.05. (**A**) Embryos stained with 2′,7′-dichlorodihydrofluorescein diacetate. (**B**) Fluorescence intensities (mean ± SEM) quantified based on grey pixel intensity.

The intracellular ROS level was significantly (P < 0.05) low in the female embryos than males (1 vs 0.54 ± 0.09) in terms of fluorescence intensities (mean ± SE) quantified on grey pixel intensity in all oxidative status of culture conditions (Fig. 5A,B).

### Sexual dimorphism in the relative expression level of developmental genes in embryos at different oxidative status

**Figure 6:**
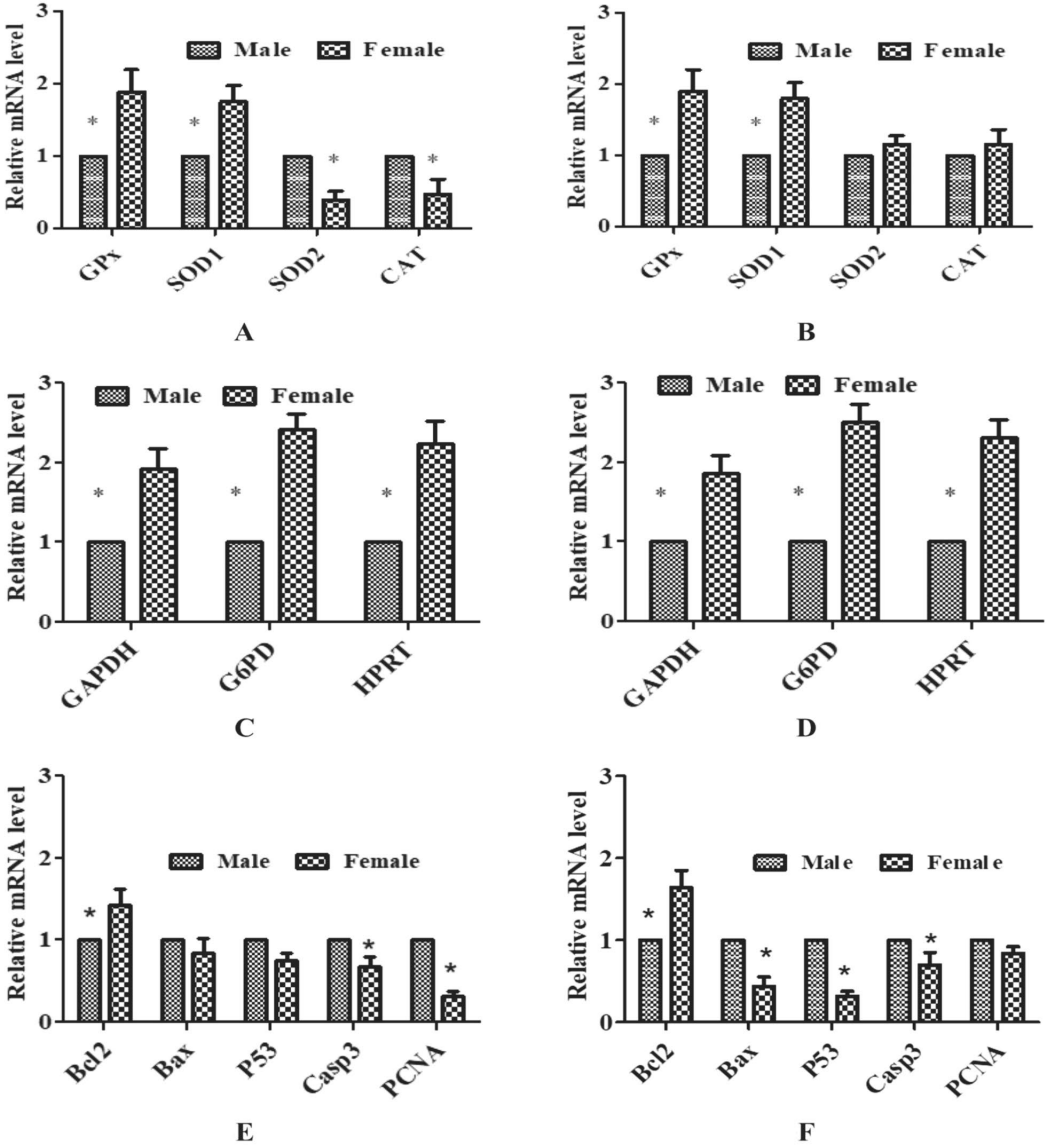
Sex-specific differences in relative expression level of developmental genes in ovine embryos at different oxidative status. (**A**) Antioxidant genes at high oxidative status. (**B**) Antioxidant genes at low oxidative status. (**C**) Glucose metabolism genes at high oxidative status. (**D**) Glucose metabolism genes at low oxidative status. (**E**) Apoptotic genes at high oxidative status. (**F**) Apoptotic genes at low oxidative status. *Significant difference between male and female at P < 0.05.

The sex-specific differences in antioxidant genes expression at high oxidative status showed significantly (P < 0.05) upregulated expression of *GPx* and *SOD1* and downregulated expression of *SOD2* and *CAT* in female embryos than males. At low oxidative status, the expression of *GPx* and *SOD1* were significantly (P < 0.05) upregulated, and the expression of *SOD2* and *CAT* were non significantly upregulated in female embryos than males. The expression of *SOD2* and *CAT* were significantly (P < 0.05) upregulated in female embryos at low oxidative status than at high (Fig. 6A,B). The sex-specific differences in glucose metabolism genes (*GAPDH, G6PD,* and *HPRT)* expression were significantly (P < 0.05) upregulated in female embryos than males in both low and high oxidative status (Fig. 6C,D). The sex-specific differences in apoptosis-related genes expression at high oxidative status showed significantly (P < 0.05) upregulated expression of *Bcl2* and downregulated expression of *Casp3* and *PCNA* in female embryos than males. However, the expression of *Bax* and *p53* were similar in both the sex of embryos**.** In turn, low oxidative status showed significant (P < 0.05) upregulated expression of *Bcl2* and downregulated expression of *Casp3, Bax,* and *p53* in female embryos than males. In contrast, the expression of *PCNA* in female embryos was upregulated and showed a significantly similar expression level as in male embryos (Fig. 6E,F).

## Discussion

Oxidative status-mediated change in oocytes polarity for sex-specific sperm fertilization to produce desired sex embryos and their subsequent development is the new biological intervention approached in the present study. The study observed that the low and high oxidative status favoured female and male embryos production and their subsequent development respectively. This is the first report on oxidative status mediated biasness of the embryonic sex ratio produced in vitro*.* Fertilization is associated with a change in resting membrane potential, referred as “fertilization potential”, and compared to the action potential of neurons^17^. The membrane potential/polarity is maintained by differences in ion concentrations between the intra and extracellular components. Change in membrane polarization and ion permeability during oocyte maturation^18^ is regulated by Na^+^/K^+^-ATPase ion transport channel^19^ and sensitive to oxidative stress^20^. Depolarization of the plasma membrane causes a shift towards positive potential and hyperpolarization negative^21^. The oocyte membrane potential undergoes a different state of polarity^17^ to facilitate the binding of sex-specific charged sperm (X or Y) during fertilization^22^ to determine the sex of embryos. The change in normal resting negative membrane potential (polarized) to a positive potential (depolarized) occurs due to the influx of more positive Na^+^ and Ca^2+^ ions from the extracellular components and ends with negative potential (repolarized). Hence, it is suggested that the oocyte membrane polarity determines the binding of sex-specific sperm. However, research finding on sex-specific sperm binding to oocytes as per their polarity is not available. Oxygen and l-carnitine mediated lowering the oxidative status of the culture system improves the developmental potential of embryos^9,14,15^. It was predicted that low oxidative status at 5% O_2_ with supplementation of l-carnitine (experiment III, group 2) would favour a significant increase in morula and blastocysts production. However, the developmental potential of embryos of this group resulted in significantly (P < 0.05) low cleavage and morula with no blastocyst. Intracellular ROS level within the physiological limit is beneficial for cells and plays an essential role in oocytes maturation and subsequent embryo development^23^. ROS generated in oocytes of group 2; experiment III got neutralized by l-carnitine from the beginning of maturation itself. As time advanced, low O_2_ (5%) availability in the surroundings and their slow diffusion in paraffin oil^24^ made ROS level below the beneficial physiological limit in the micromilieu. That, in turn, might have negatively affected the metabolic pathways suitable for embryonic development. Marginal increase in intracellular ROS in the matured oocytes is useful for subsequent developmental stages of embryos, and ROS generation in developmental stages is important for blastocyst formation^25^. During IVEP, low ROS improves the developmental potential of embryos, and high ROS has deleterious effects on embryonic development resulting in apoptosis^9,11^. Hence, it was concluded that though low ROS improved the developmental potential of embryos, significantly low ROS below the beneficial physiological limit hindered development. This study sought to modify the oocyte’s membrane polarity, modulating oxidative status of the culture conditions for sex-specific sperm fertilization to get desired sex embryos. Oxygen (20 vs. 5%) mediated oxidative status of culture condition could not result in significant (P < 0.05) embryonic sex bias. The majority of the embryos produced at both the O_2_ (20 and 5%) (experiment I) were male-biased. However, the female embryos percentage was significantly (P < 0.05) high at 5% O_2_ than at 20%. Oxygen-mediated oxidative stress-induced embryonic sex ratio of this study was in agreement with the report suggesting high O_2_ has a greater detrimental effect on female embryos than males. Male embryos develop faster than females in vitro (high O_2_) and the reverse is in vivo (low O_2_)^3^. The embryos produced at 5% O_2_ have low intracellular ROS than at 20%^9^. Hence, it was expected that 5% O_2_ would favour a significant increase in female embryos production. However, the majority of the embryos produced at 5% O_2_ were biased towards males like they were biased at 20% O_2_. Oxygen diffusion through oil is important during in vitro oocytes and embryos culture. However, the oil prevents the diffusion of O_2_ to the cells. Thus, the O_2_ availability to micro drops under paraffin oil gets reduced due to slow diffusion through oil and geometry of the drop^24^.

It was speculated that the intracellular ROS level during oocyte maturation at both the O_2_ (20 and 5% O_2_) concentrations was initially due to ROS production by oocytes. As the time advanced, the ROS level in oocytes cultured at high (20%) O_2_ tension was increased by diffusion of more available surrounding O_2_ through paraffin than the low surrounding O_2_ (5%). Hence, the time by which the O_2_ concentration mediated differences in ROS level should influence the sex-specific sperm fertilization; by that time majority of the Y sperms have been fertilized, and significantly (P < 0.05) more male embryos were produced. It can also be explained that adequate ROS must have been generated in the culture medium by the time taken to reach 5% O_2_ in micromilieu. Hence, ROS-mediated alkalinity of the medium must have kept oocytes in the repolarized state (negatively charged) to favour fertilization of positively charged Y sperm resulting in significantly (P < 0.05) more male embryos. There was a non-significant increase in intracellular Na^+^ and Ca^2+^ ions in the oocytes matured at 5%O_2_ than at 20%, with no change in K^+^ ion concentration. Significantly (P < 0.05) more male embryos production at both the O_2_ concentrations suggested that because of non-significant change in intracellular positive ions, the majority of the oocytes matured in these O_2_ (20 and 5%) concentrations were in the repolarized state and favoured Y sperm fertilization resulting male embryos. However, because of a non-significant increase in intracellular positive ions in oocytes matured at 5% O_2_, a greater number of oocytes were in a depolarized state and fertilized with negatively charged X sperm resulting in significantly (P < 0.05) more female embryos than at 20% O_2_. In the same fashion, more oocytes were in a repolarized state when matured at 20%O_2_ and fertilized with Y sperm resulting in significantly (P < 0.05) more male embryos than at 5% O_2_. It was predicted that low ROS at 5% O_2_ would favour a significant increase in female embryos production. However, by the time low ROS-mediated pH of the medium increased the intracellular positive ions of oocytes to retain in a depolarized state for X sperm-specific fertilization, the majority of Y sperms got fertilized, resulting in considerably more male embryos. Low O_2_ (5%) affects oxidative phosphorylation resulting in an increase in intracellular lactic acid production from pyruvate, which lowered the pH of the culture medium, i.e., acidic as compared to 20% O_2_ and favoured fertilization of X sperm to produce more female embryos^26^. The oocytes matured with l-carnitine have significantly (P < 0.5) more Na^+^ and Ca^2+^ ions than matured without l-carnitine with no change in K^+^ ion. The influx of more positive ions in the oocytes matured with l-carnitine has kept the oocytes in the depolarized state to favour X sperm fertilization, resulting in significantly more female embryos. In contrast, the repolarized membrane oocytes matured without l-carnitine were receptive to Y sperms and produced significantly more male embryos. The sex-specific embryo production by l-carnitine supplementation in maturation and culture media of this study has been filed for an Indian patent with application number: 202041051123 TEMP/E-1/56809/2020-CHE dt. 24. 11. 2020.

l-Carnitine-mediated lowering of intracellular ROS in oocytes and embryos has improved the developmental potential of embryos^14,15^. Sodium channels remain active up to the full maturity of the oocytes^27^; however, K^+^ ion permeability progressively decreases along with maturation^28^, and activity of Ca^2+^ channels decreases in matured oocytes^29^. Hence, it can be suggested that due to the progressive decrease in permeability of K^+^ ions in matured oocytes, its concentration was not changed in both groups (experiments I and II). The permeability of Na^+^ and K^+^ ions must have been influenced by the slow Ca^2+^ channel that opens slower and remains open much longer time than Na^+^ channels in oocytes because of its smooth muscle property^10^. The opening of slow Ca^2+^ channels accounts for the prolonged plateau action potentials in smooth muscle fibers, and the repolarization is delayed; hence, the concentration of K^+^ ion in both groups was not changed significantly. Increased intracellular H^+^ ion concentration (acidification) results in membrane depolarization, whereas a decreased intracellular H^+^ ion concentration (alkalinization) results in hyperpolarization of the membrane. Membrane potential is markedly depolarized by decreasing the pH (acidic) of the external solution^30^. There is a production of more ROS at high O_2_ concentration during IVEP^9^. Hence, it is justified to say that the oocytes matured at high O_2_ (experiment I) and matured without l-carnitine (experiment II) produced more ROS, making the maturation medium alkaline and kept the oocytes in the repolarized state to favour Y sperm fertilization to produce significantly (P < 0.05) more male embryos. Subsequently, maturation of oocytes at low O_2_ (5%) (experiment I) and supplementation of l-carnitine in the medium (experiment II) significantly reduced ROS production and elicited acidification of medium that favoured the X sperm fertilization to the depolarized oocytes to produce more females. The explanation for the depolarization elicited by acidification would be the entry of positively charged ions^31^, which has already been observed in this experiment. There is a difference in pH-mediated X and Y sperm motility and viability. An alkaline seminal fluid favours males and an acidic vagina favours females^32^. Both X and Y-bearing sperm survive longer in the slightly alkaline environment, but Y sperm is more labile under acidic conditions. In contrast, X sperm would survive longer in an acidic medium^33^. Hence, it can be concluded that the sex ratio of the embryos produced at different oxidative status must have influenced the pH-mediated ionic exchange of the oocytes membrane to change their polarity for subsequent fertilization of respectively charged spermatozoa to result in sex-specific embryos. As the developmental stages of embryos advanced, it was observed that the low and high oxidative status of culture conditions were suitable for the developmental potential of female and male embryos respectively. Oxidative status-mediated embryonic sex biasness of this study summarized that O_2_-mediated oxidative status of culture condition could not result in significantly more female embryos as resulted in l-carnitine supplementation in the media. l-Carnitine neutralized the ROS from the beginning of maturation to modulate the pH of medium for the subsequent depolarized state of oocytes to favour X sperm-specific fertilization to produce more female embryos. Whereas, neutralization of ROS from the beginning was not possible in oocytes matured at 5% O_2_ because of the time taken to come down from atmospheric O_2_ concentration of CO_2_ incubator to 5% and was also influenced by the slow diffusion of O_2_ in oil and geometry of drop^24^. Therefore, the main critical factor for oxidative status mediated embryonic sex biasness was the fertilization of sex-specific sperms to the oocytes as per their polarity through alteration in the pH of the medium, modulating the intracellular positive ions.

Despite the different levels of oxidative status, the intracellular ROS level was significantly (P < 0.05) low in female embryos than males. Low intracellular ROS in female embryos agreed with a few studies^34,35^. Upregulated expression of *GPx* in female embryos at both the oxidative status might be due to significantly (P < 0.05) low intracellular ROS mediated antioxidant capacity of female embryos. Mitochondrial SOD (*SOD2*) is an oxidative stress indicator in embryos, and its expression is culture condition-dependent^36^. Cytoplasmic SOD (*SOD1*) expression is considered as the first line of defense against ROS and is essential together with GPx to protect embryos from oxidative damage^36^. In this study, there was low intracellular ROS in female embryos with upregulated expression of *GPx*. As a result, ATP synthesis would be unaffected in female embryos utilizing a large quantity of O_2_, resulting in low ROS formation, thereby showing downregulated expression of *SOD2*^37^. However, upregulated expression of *SOD1* despite downregulation of *SOD2* in the female embryos at high oxidative status suggested that there might be some other cellular or transcription factors influencing the expression level of *SOD1,* keeping female embryos more stressful than males during IVEP, that differentiates the developmental kinetics of sexed embryos. Over-expression of *CAT* prevents mitochondrial ROS production^38^. As a result, low intracellular ROS in female embryos showed downregulated expression of *CAT*. There is no such literature available to compare our result for the sexual dimorphism in the embryonic antioxidant genes expression. However, available literature on clinical studies reported that females appear to be less susceptible to oxidative stress under physiological conditions due to the antioxidant properties of estrogen and gender differences in NADPH-oxidase activity^39^. GPx is the only antioxidant enzyme that consistently showed a gender biasness across several studies^40^. Estrogen acts as a potential antioxidant in females, so less GPx is required in females than males^41^. Hence, the antioxidant action of estrogen could be the reason for the gender-specific differences in *GPx* expression. However, the present study could not prove the antioxidant properties of estrogen properly for upregulated expression of *GPx* in female embryos*.* Significant (P < 0.05) downregulation of *SOD2* and *CAT* in female embryos than in males at high oxidative status was in agreement with the clinical finding suggesting mitochondrial ROS production in females is significantly lower than that of males^42^. *SOD* and *GPx* expression are estrogen-dependent, whereas *CAT* expression is not^43^, and that might be the reason for no difference in catalase activity levels between males and females in some studies^44^. The antioxidant enzyme activity is strongly regulated by estrogen in females, and total SOD activity is more in females than in males^45^. Antioxidant-mediated upregulated expression of *GPX* and *SOD2* in ovine embryos is evident^46^. Hence, overexpression of these antioxidant genes at low oxidative status in female embryos enhanced the antioxidant mediated defense against oxidative stress, resulting in better female embryos development.

Glucose metabolism genes (*G6PD* and *HPRT*) are X chromosome-linked^47^. Since female embryos contain double the dose of the X chromosome than males, the expression of these genes showed upregulated expression in female embryos. G6PD is responsible for sex-specific differences in embryonic metabolism and is highly expressed in female embryos^48,49^. In contrast, *G6PD* and phosphoglycerate kinase (*PGK*) expression were similar in both the sex of embryos^48^*.* Most of the X-linked genes have significantly higher expression in female embryos than males and are regulated differently between the sex creating sexual dimorphism in developmental kinetics^50^. The significant (P < 0.05) upregulated expression of most of the X-linked genes in female embryos is suggestive of partial X-chromosome inactivation (XCI). XCI is essential for female embryogenesis for equal dosages of X-chromosome-linked genes in both the sex that leads to the silencing of one X-chromosome in female^51^. The level of *G6PD* was significantly higher in female embryos than males, but *HPRT* levels were similar in both sex^50^. The *G6PD* and *HPRT,* subjected to dosage compensation, should not have significantly different expression levels between the sex when X-inactive specific transcript (XIST) expression is detected in female embryos. The process of XCI is controlled by the X inactivation center (XIC) and the resident gene XIST. The expression of genes located on the X chromosome at different distances from the XIC. *HPRT* is closer to the XIC than *G6PD* on the X chromosome^52^. The expression of *G6PD* and *HPRT* are similar between males and females of two cells embryos before embryonic genome activation but higher in females than males at the blastocyst stage^7^. In the present study expression level of *G6PD* was higher in female embryos than males; however, the developmental potential of female embryos was less than males at high oxidative status. This finding agreed with the report suggesting the sex-specific differences in embryonic *G6PD* expression and female embryos have a lower developmental rate than males with an increase in *G6PD* expression^53^. In contrast, the embryos with a high *G6PD* expression have more developmental competence than those with low *G6PD*^54^. G6PD is the important enzyme of the pentose phosphate pathway and is essential for generating NADPH. NADPH is responsible to maintain glutathione during cellular oxidative stress to scavenge ROS. Hence, G6PD plays a crucial role in protecting embryos from ROS-induced apoptosis affecting the developmental potential of embryos^55^. There was no study to compare our result with sex-specific differences in expression of *GAPDH* (enzyme for glycolysis pathway), an autosomal gene in embryos. However, phosphate glycerate kinase (PGK) enzyme responsible for the glycolysis pathway was upregulated in female embryos^49^ which can agree with our finding of upregulated expression of *GAPDH* in female embryos. Most X-linked genes display not only sex-related transcriptional differences but are also involved in regulating the autosomal gene expression in preimplantation embryos^56^. *GAPDH* showed significantly higher expression in females than males^57^ which was in accordance with our result showing upregulated expression in female embryos than males. Hence, it can be suggested that high oxidative status affected severely the double gene doses chromatin of X chromosome in female embryos than males. Thus, ROS-induced damage or inactivation of the X chromosome hinders the developmental potential of female embryos at high oxidative status.

*Bcl2* expression was significantly (P < 0.5) upregulated in female embryos at both the oxidative status suggesting them more protected from apoptosis, that agreed to the report suggesting female embryos have low intracellular ROS than males^34^ and also observed in the present study. However, in contrast, female embryos are more prone to apoptosis than males because several proapoptotic genes are upregulated^58^. *Bcl2* prevents apoptosis of embryos and the transcriptional level of *Bcl2* and *Bax* is correlated with the developmental competence of oocytes^59^. The present study reflected the better developmental potential of male embryos over females at high oxidative status, which might be due to ROS-induced destruction in cellular metabolic pathways that must not be favouring the developmental potential of female embryos. However, low oxidative status protected the female embryos from apoptosis with supportive alteration in the expression of apoptosis-related genes. Sexual dimorphism in the developmental potential of embryos at high oxidative status favouring male embryos suggested that transcription factors present in the Y-chromosome must be influencing their development after fertilization^60^. Though the expression of *Bax* and *P53* were not significantly (P < 0.5) different in both the sex at high oxidative status, low oxidative status downregulated the expression of both the genes in female embryos suggesting protection from ROS-induced apoptosis. *p53,* an apoptosis regulator, is correlated with oxidative stress response and is critical during in vitro embryo development. A high *p53* is responsible for apoptosis. Reduction in ROS depletes *p53* in embryos and protects embryos from apoptosis^61^. *p53* is activated by oxidative stress and causes developmental arrest, whereas suppression of *p53* may not support embryo development due to ROS-induced DNA damage^62^. In the present experiment, high oxidative status upregulated the expression of *p53* in female embryos. Whereas, low oxidative status downregulated the expression of *p53*, in turn, reduced ROS-induced DNA damage, thus, improved the developmental potential of female embryos, and has been confirmed by upregulated expression of *PCNA* in those embryos. *PCNA* is responsible for cell death or survival. A high level of *PCNA* in the presence and absence of *p53* is responsible for the repairing of DNA damage and DNA replication, respectively. In the state of irreparable DNA damage, PCNA drives the cell to apoptosis^63^. Low oxidative stress created a suitable micromilieu for nuclear programming in female embryos, improving their developmental potential and upregulated *PCNA* expression. Upregulated expression of *Casp3* induces developmental arrest of embryos^64^. The expression level of *Casp3* of this study was significantly (P < 0.05) lower in female embryos than males in both oxidative stress. Low intracellular ROS level in female embryos than males might have influenced the downregulated expression of *Casp3*. l-Carnitine supplementation in the media has shown no change in the expression level of *Casp3* at the blastocyst stage^15^. The developmental potential of female embryos was affected at high oxidative status despite low *Casp3* expression in them. Hence, it was speculated that there must be some other factors affecting the developmental potential of female embryos. ROS-mediated inactivation of energy metabolism in female embryos at high oxidative stress must have induced the downregulated expression of *Casp3* and *PCNA*. However, low oxidative stress improved the energy metabolism in those embryos and helped to improve their developmental potential. The female embryos at low oxidative stress have gone less detrimental, resulting in significantly (P < 0.05) more percentage. Therefore, it is concluded that the expression of transcripts resulted in sexual dimorphism in developmental kinetics and epigenetics in preimplantation embryos during development^50^. However, the main critical factor for oxidative status mediated embryonic sex biasness was the fertilization of sex-specific sperms to the oocytes as per their polarity through alteration in the pH of the medium, modulating the intracellular positive ions.

## Materials and methods

All the methods were performed in accordance with relevant guidelines and regulations. Institutional Animal Ethics Committee, ICAR-National Institute of Animal Nutrition and Physiology approved the present study (No. NIANP/IAEC/1/2017).

### In vitro ovine embryo production

Embryos were produced from oocytes of slaughterhouse ovaries through in vitro maturation, fertilization, and culture^9^. Oocytes were aspirated from follicles (2–6 mm) in collection medium {(TCM-199 (glutamine added) + BSA (3 mg/ml) + 5% FBS + heparin (10 μg/ml)**}** and matured with TCM-199 (glutamine added) + 10% FBS + BSA (3 mg/ml) + pyruvate (4 mM) + gentamicin (50 μg/ml) + FSH (5 μg/ml) + LH (5 μg/ml) + estradiol (1 μg/ml) + EGF (20 ng/ml) for 27 h at 5% CO_2_, and 38.5 °C at different O_2_ concentrations with/without l-Carnitine (10 mM). Fresh semen collected by electro-ejaculator was washed with modified synthetic oviductal fluid (MSOF) + heparin (10 μg/ml) + pyruvate (1 mM)} by centrifuging at 400 g for 5 min. Supernatant was removed and pellet was reconstituted in MSOF + fatty acid-free BSA (4 mg/ml) + heparin (10 μg/ml) + pyruvate (1 mM) + BME (100 ×) (1%) + MEM (50 ×) (1%), and adjusted to 2–3 × 10^6^ sperms/ml. Matured oocytes were inseminated with processed spermatozoa for 18–20 h. Following co-incubation, presumptive zygotes were cultured in TCM-199 (glutamine added) + 20% FBS + BSA (3 mg/ml) + pyruvate (4 mM) + gentamicin (50 μg/ml) + BME (100 ×) (1%) + MEM (50 ×) (1%) at different O_2_ concentrations with/without l-Carnitine (10 mM) to get developmental stages (cleavage to blastocyst) of embryos. Cleavage rates and blastocyst were recorded on day 2 and 7 respectively (day 0 = day of IVF).

### Polarity of the matured oocytes

The membrane potential/polarity of oocytes are maintained by disparities in essential ions i.e., Na^+^, K^+^, and Ca^2+^ concentrations across the membrane. An increase in intracellular positive ions makes the membrane positive i.e., depolarized and a decrease in positive ions keeps negative i.e., repolarized. The intracellular ions concentrations of the matured oocytes of experiments I and II (significantly producing sex-specific embryos) were estimated through inductively coupled plasma optical emission spectroscopy (ICP-OES) analytical technique (Optima 8000, Perkin Elmer) as per the manufacturer’s guideline. Oocytes were treated with 0.25% Trypsin–EDTA to remove cumulus cells and digested with Proteinase K (100 mg/ml) at 56 °C/2 h with inactivation at 95 °C/10 min and cooled in ice. The mixture was vortexed and centrifuged at 2000*g/*10 min. The supernatant was used to estimate Na^+^, K^+^, and Ca^2+^ ions concentration. Oocyte’s membrane polarity was calculated by Goldman–Hodgkin–Katz equation as EMF (millivolts) = − 61 log concentration of positive ions inside/outside and concluded based on intracellular positive ions concentrations.

### Preparation of embryos for sex determination

The embryos (cleaved to blastocyst) were exposed to 0.2% protease to remove zona pellucida because attached sperms may give the wrong sex determination. Protease exposure was inactivated by TCM-199 + 25% FBS. Few blastomeres were washed by TE buffer (10 mM) and digested with Proteinase K (100 mg/ml) at 56 °C/2 h with inactivation at 95 °C/10 min and cooled in ice. The mixture was centrifuged at 2000*g/*10 min and the supernatant (3–5 μl) was used in PCR for sexing^65^. Rest of the blastomeres were stored in Trizol for gene expression study.

### Preparation of blood as positive control for PCR

Single-step DNA isolation from sex-specific (male and female) blood through alkaline lysis was approached to use for sex determination^65^. Blood (5 μl) was mixed with 50 mM NaOH (50 μl) and incubated at 37 °C/10 min followed by boiling at 95 °C/10 min. The incubation was terminated by 1 M Tris HCl (5 μl). The mixture was centrifuged at 2000*g/*10 min and the supernatant (2–3 μl) (1:4 dilution) was used in PCR as the positive sample for the primers used for the sexing of embryos.

### Sex determination of blood and embryos

The sexing of the blood and embryos was carried out by the PCR-based amplification of *SRY* and *AMEL* genes present in gDNA^65^. PCR for *SRY* gene amplification was with an initial denaturation at 96 °C/6 min and 6 cycles of touchdown PCR at 95 °C/30 s, 63–58 °C (− 1 °C/cycle)/45 s, and 72 °C/45 s followed by 34 cycles of 95 °C/30 s, 59 °C/45 s, and 72 °C/45 s. *AMEL* gene amplification was with denaturation at 96 °C/6 min and 8 cycles of touchdown PCR at 95 °C/45 s, 70–63 °C (− 1 °C/cycle)/60 s, and 72 °C/75 s followed by 34 cycles of 95 °C/45 s, 64 °C/60 s, and 72 °C/75 s. The final extension for both the genes was at 72 °C/10 min. The gene-specific primers for sexing were designed from NCBI, Primer Blast (Table 3).

**Table 3:** Primers used for sex determination of embryos and gene expression study.

Genes	Primer sequences (5′–3′)	Product size
*AMEL*	FP: CCGCCCAGCAGCCCTTCC; RP: CCCGCTTGGTCTTGTCTGTTGC	Male: 243 and 198 bp Female: 243 bp
*SRY*	FP: GCGCAAACGATCAGCGTGAA; RP: TCGTATCCCAGCTGCTTGCT	Male :169 bp Female: not amplified
*ACTB*	FP: CCTGGCACCTAGCACAATGA; RP: TGGAAGGTGAACAGTGCGAG	102 bp
*GPx*	FP: CGTGCAACCAGTTTGGGCAT; RP: GATGCGCCTTCTCGCCATTC	141 bp
*SOD1 (Cu/Zn-SOD)*	FP: CCACTTCGAGGCAAAGGGAGA; RP: CCTTTGGCCCACCGTGTTTT	167 bp
*SOD2 (Mn-SOD)*	FP: CCGTCAGCCTTACACCAAGT; RP: CAAGCCACGCTCAGAAACAC	112 bp
*CAT*	FP: GCCTGTGTGAGAACATTGCG; RP: TCCAAAAGAGCCTGGATGCG	121 bp
*GAPDH*	FP: ATGGGCGTGAACCACGAGAA; RP: ATGGCGTGGACAGTGGTCAT	146 bp
*G6PD*	FP: TCGGAGCTCGACCTGACCTA; RP: GCCTTCTCGCGTTCGATGTG	176 bp
HPRT	FP: AGCCCCAGCGTGGTGATTAG; RP: ATCTCGAGCCAGTCGTTCGG	144 bp
*BCL2*	FP: ATGACTTCTCTCGGCGCTAC; RP: CTCCACACACATGACCCCTC	176 bp
*BAX*	FP: CATGGAGCTGCAGAGGATGA; RP: GTTGAAGTTGCCGTCGGAAA	100 bp
*P53*	FP: CTGAGTGCACCACCATCCAC; RP: TCTCCCAGGACAGGCACAAA	161 bp
*CASP3*	FP: ACCTCACGGAAACCTTCACGA; RP: ACCATGGCTTAGAAGCACGC	149 bp
*PCNA*	FP: AGCCACTCCACTGTCTCCTACA; RP: TCATCCTCGATCTTGGGAGCC	123 bp

### Intracellular ROS levels in sex-specific embryos

Before sex determination, intracellular ROS was estimated in the embryos (experiments I and II) using 2′,7′-dichlorodihydrofluorescein diacetate (DCHFDA) ^14^. Embryos were washed twice in PBS + polyvinyl pyrrolidone (PVP) (0.5%) (wt/vol) and fixed with 4% paraformaldehyde and placed in 10 µM DCHFDA (50 µl)/15 min. Finally, the embryos were washed three times by PBS + PVP (0.5%), carefully mounted on a glass slide, and covered with a coverslip. The fluorescence intensity was observed under an epifluorescence microscope (Euromex, Holland) and analyzed by grey pixel intensity using Image J software (NIH, USA), normalizing male embryos as 1. After ROS determination, embryos were processed for sex determination.

### Relative expression level of developmental genes in sex-specific embryos

The mRNA abundance of genes was analyzed by real-time quantitative PCR (qPCR). The sex-specific embryos produced in experiment II (significantly producing sex-specific embryos) were chosen for the gene expression study. RNA was isolated from a pool of sex-specific embryos (blastocysts) (n = 10), cDNA was synthesized, and qPCR was carried out using gene-specific primers designed from NCBI, Primer Blast (Table 3).

### Total RNA isolation from embryos and cDNA synthesis

Total RNA was isolated by Trizol (Invitrogen, Life Technologies, USA)^66^. TRIzol (200 μl) was added to the embryos, mixed, and incubated at room temperature/10 min. Chloroform (50 μl) was added, mixed, and incubated at room temperature/10 min. The mixture was centrifuged at 12,000*g*/15 min at 4 °C, and the upper aqueous phase was collected. Acrylamide (20 µg) and isopropanol (100 μl) were added to the aqueous phase, mixed, and incubated on ice for 1 h. The tubes were centrifuged at 12,000*g*/10 min at 4 °C after incubation, and the supernatant was discarded. The pellet was washed with 75% ethanol (150 μl) by centrifuging at 7500*g*/5 min at 4 °C, and the supernatant was discarded. The pellet was dried at 37 °C/10 min and dissolved in DEPC water. The dissolved pellet was incubated at 55–60 °C/10 min with slight shaking in-between incubation. The gDNA contamination was removed using the TURBO DNA-*free™* kit (Ambion, Life Technologies, USA). Isolated RNA was used as the template for the first-strand synthesis using cDNA synthesis kit (Thermo Scientific, Massachusetts, USA).

### Real-time quantitative PCR (qPCR)

The qPCR was carried out for three different sets of embryos in duplicate using step one plus qPCR system (Applied Biosystem, USA)^64^. The relative quantification method was used to analyze the gene expression level using β-actin as the reference gene. The qPCR was performed in 10 μl reaction containing SYBR Fast 2× master mix (KAPA Biosystems, Wilmington, USA), gene-specific forward and reverse primers, cDNA, and final volume was adjusted with nuclease-free water. The qPCR was with an initial denaturation at 95 °C/2 min with 40 cycles of 95 °C/3 s and 60 °C/30 s. The melting curve analysis was carried out to confirm the qPCR specificity. Ct (threshold cycle for target amplification) values were analyzed using the 2^−∆∆Ct^ (normalized expression ratio) method.

### Confirmation of PCR and qPCR amplicons

The PCR amplicons for sex determination and qPCR amplicons of genes were confirmed by ethidium bromide (0.5 μg/ml) stained 2.5% agarose gel electrophoresis.

### Experimental design

Three different experiments were designed to create different levels of oxidative status, and the embryos were produced. All the embryos (cleavage to blastocyst) produced were used for sex determination. The sex ratio of the embryos was calculated as total embryos and transferable embryos.

### Experiment I

Immature oocytes were matured, fertilized, and cultured to produce embryos at different O_2_ concentrations (20 and 5%). Embryos (cleavage to blastocyst) produced were used for sexing to calculate the sex ratio.

### Experiment II

Immature oocytes of the first group were matured, fertilized, and cultured without l-carnitine. Whereas, oocytes of the second group were matured and cultured with l-carnitine (10 mM) but fertilized without l-Carnitine. The oxygen concentration of this experiment was set at 20%. Embryos produced were used for sexing to calculate the sex ratio.

### Experiment III

Immature oocytes were matured and cultured at different O_2_ concentrations (atmospheric and 5%) with l-carnitine (10 mM) but fertilized without l-carnitine. The embryos of the first group were produced in a CO_2_ incubator with no provision of O_2_ control hence, considered that the embryo production was at atmospheric O_2_. Whereas, the embryos of the second group were produced in CO_2_ incubator with O_2_ control and was set at 5%.

### Statistical analysis

The results are expressed in mean ± SEM. Statistical analysis was carried out using GraphPad Prism5, San Diego, USA. Sex ratio of embryos, the sex-specific difference in ROS, intracellular ions concentration, and relative gene expression level in embryos were compared by student’s ‘T’ test. P < 0.05 was considered as significant.

### Ethics approval

The research protocol was approved by the Institutional Animal Ethics Committee (IAEC) of the ICAR-National Institute of Animal Nutrition and Physiology with approval number NIANP/IAEC/1/2017 dated: 25.3.2017.

## Supplementary Information

Supplementary Information.

## Data Availability

All materials are available on request.
