# Supplementary material for: In vitro production of desired sex ovine embryos modulating polarity of oocytes for sex-specific sperm binding during fertilization
Source: Sci Rep. 2022 Apr 7;12:5845. doi: 10.1038/s41598-022-09895-2 (PMC8991187; doi:10.1038/s41598-022-09895-2)
Supplement: Supplementary file 1 — Supplementary Information. [file 41598_2022_9895_MOESM1_ESM.pdf]

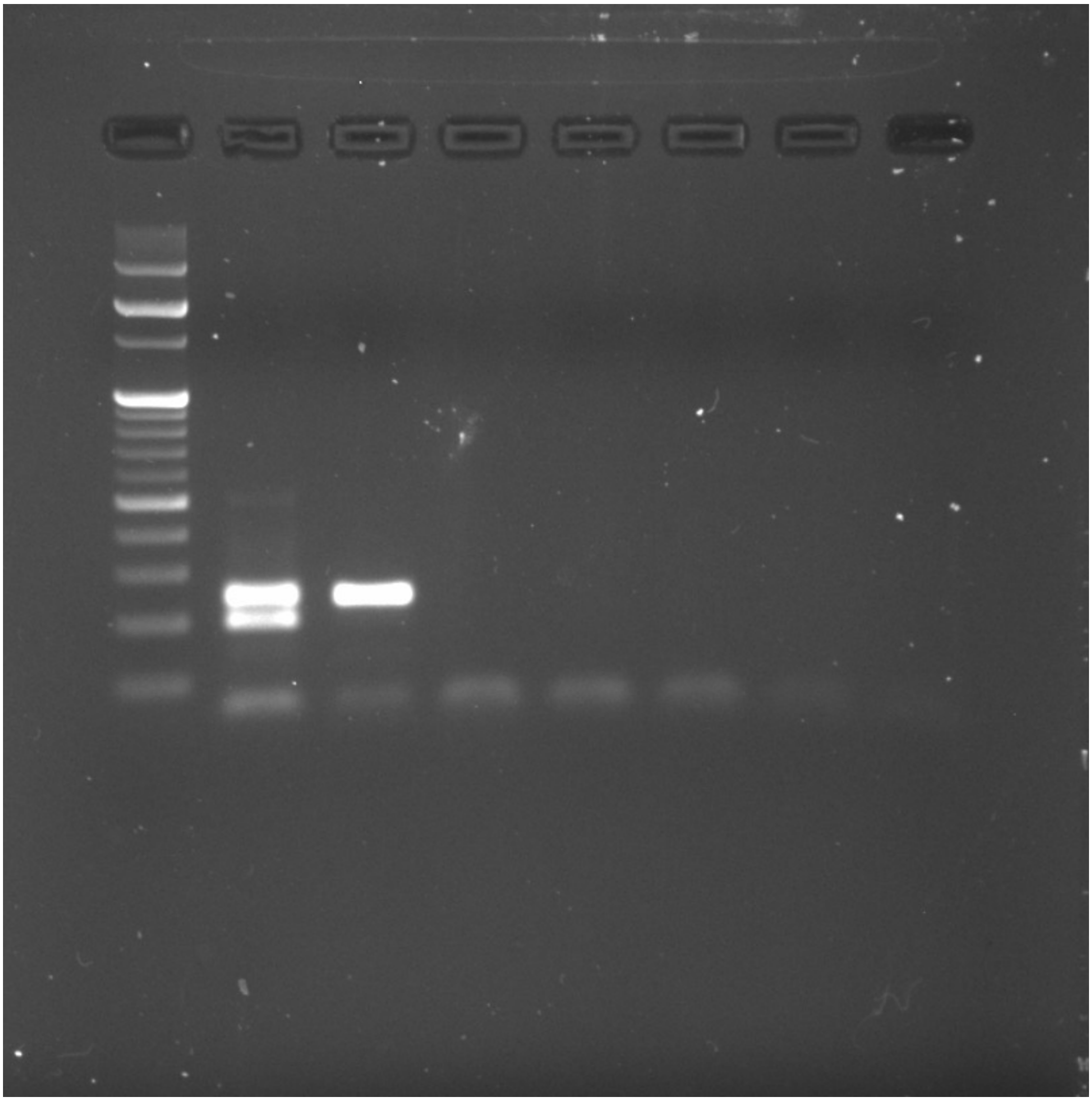

Original Gel photograph for AMEL in Figure 2

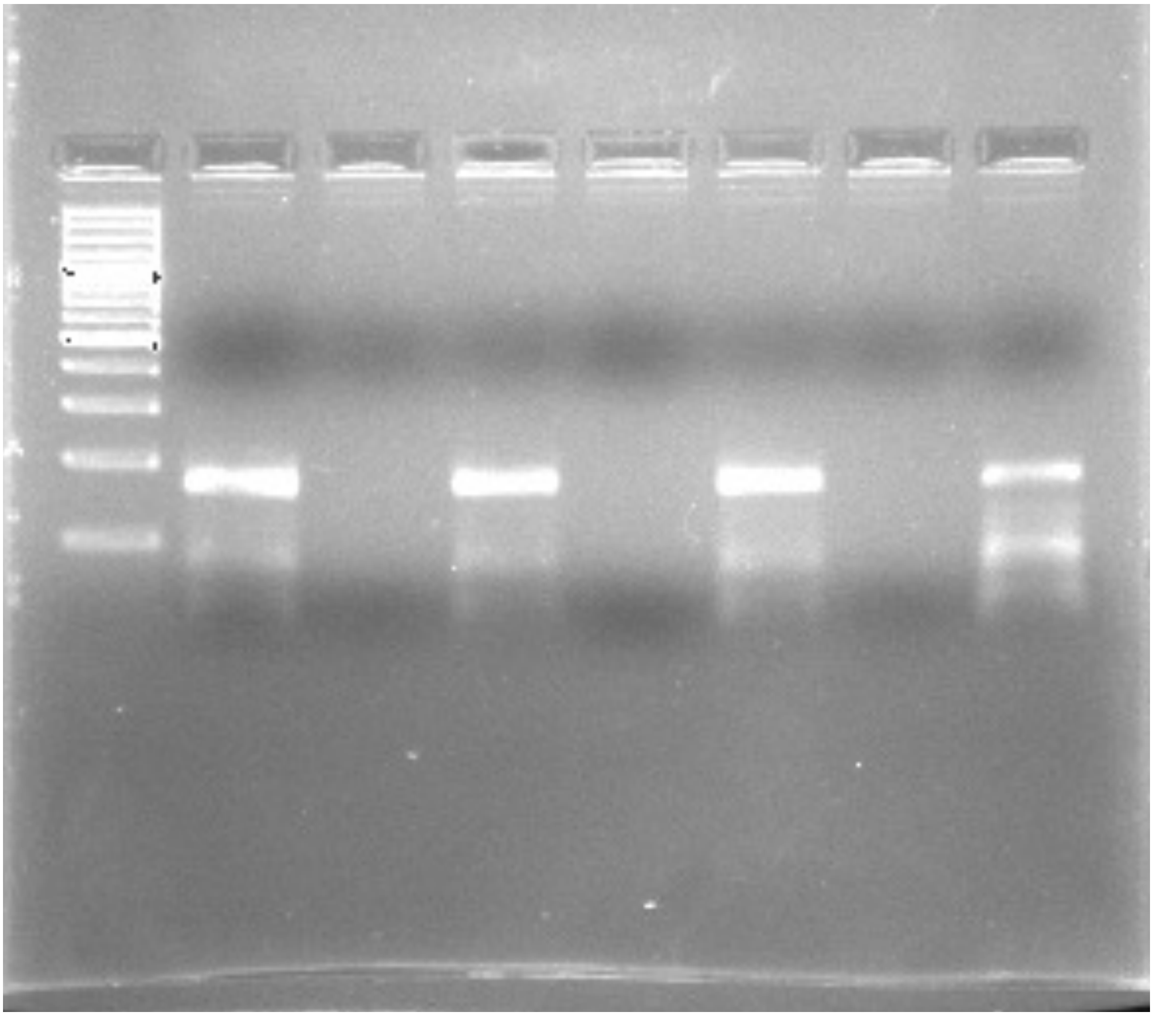

Original Gel photograph for SRY in Figure 2

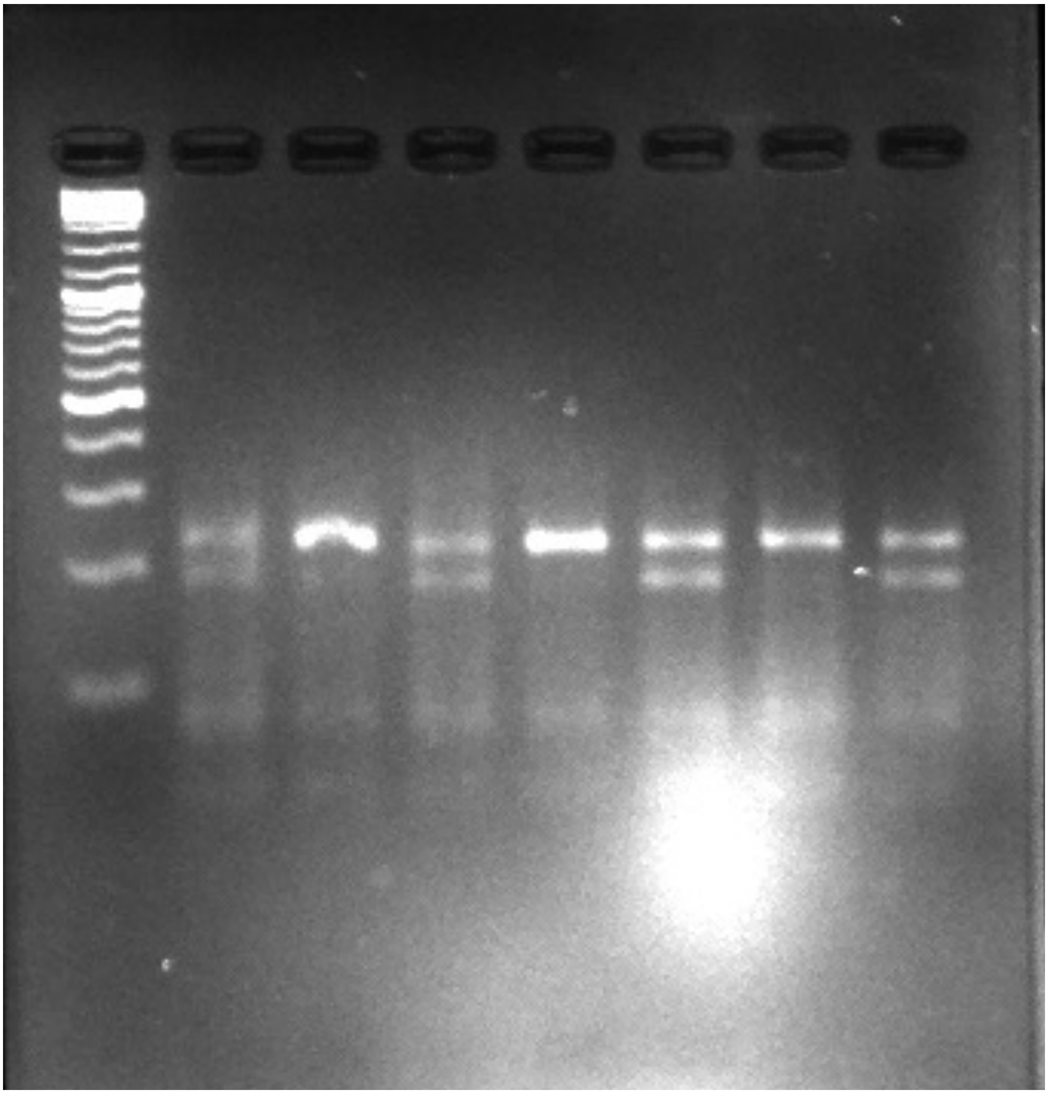

Original Gel photograph for AMEL in Figure 3

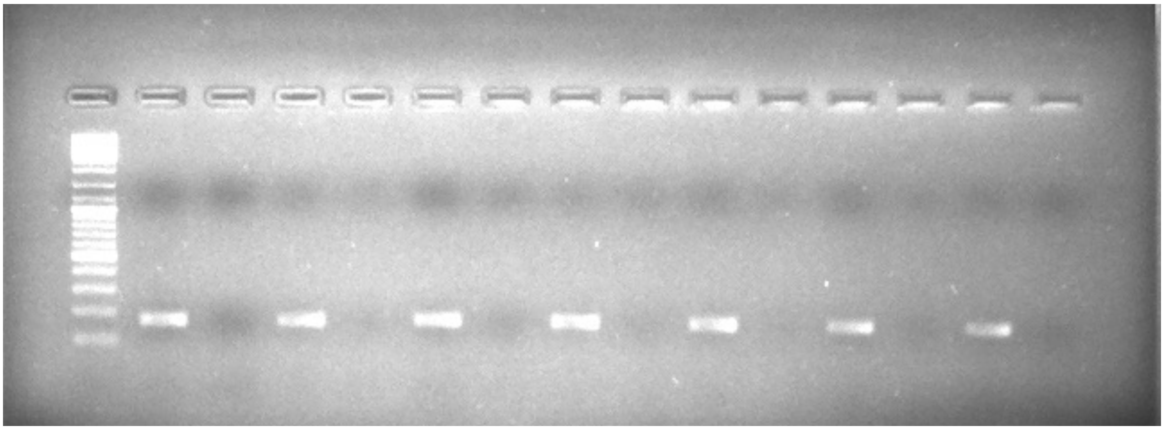

Original Gel photograph for SRY in Figure 3
